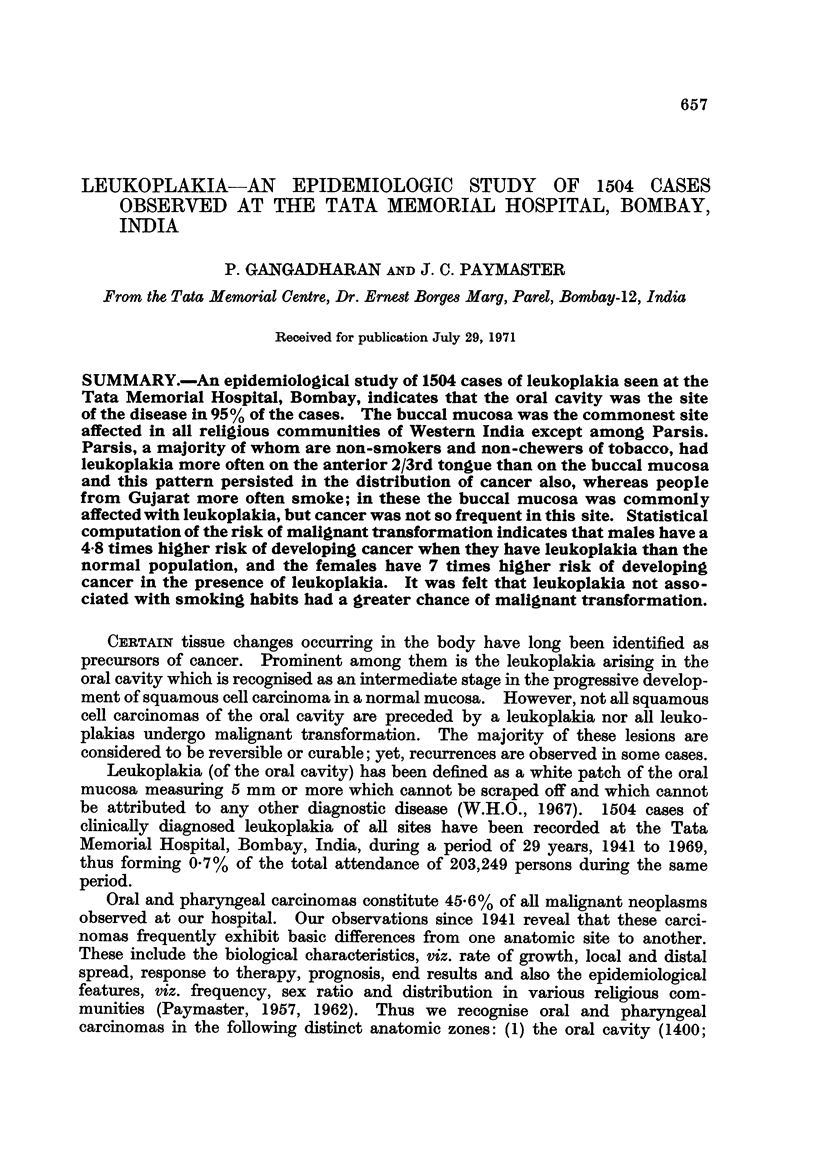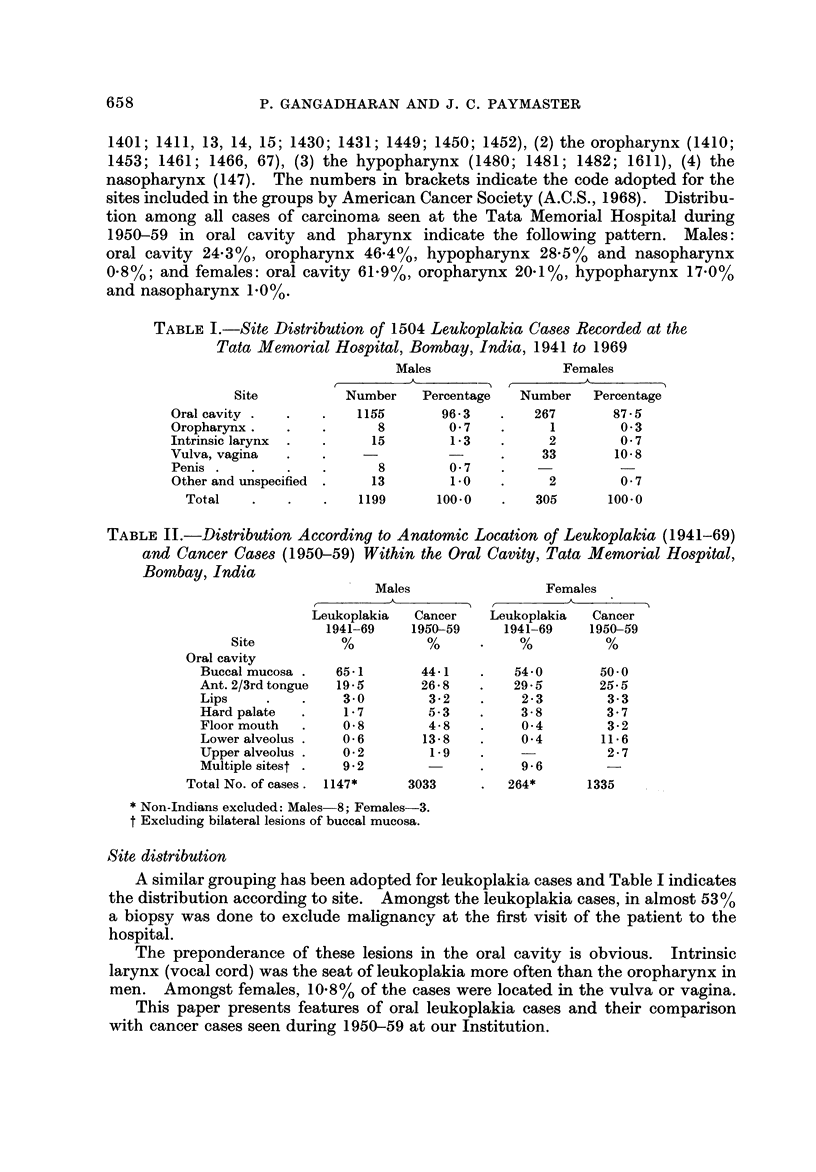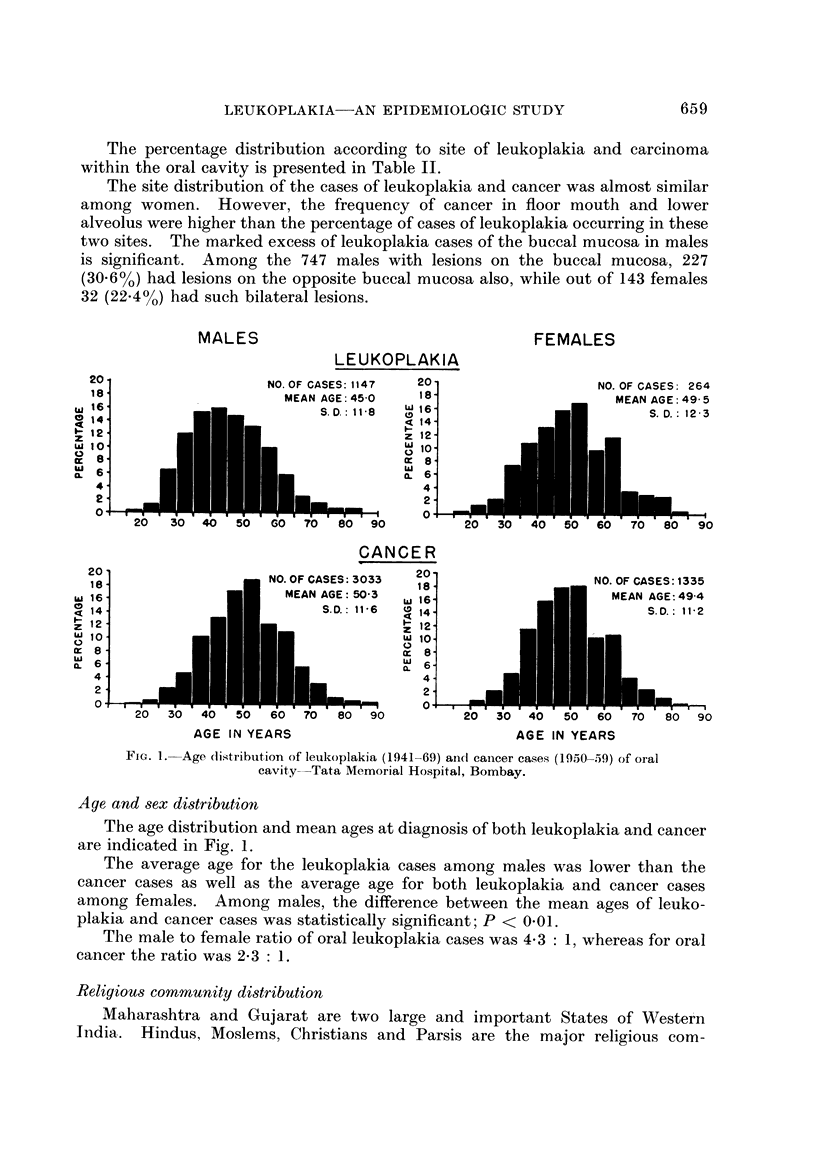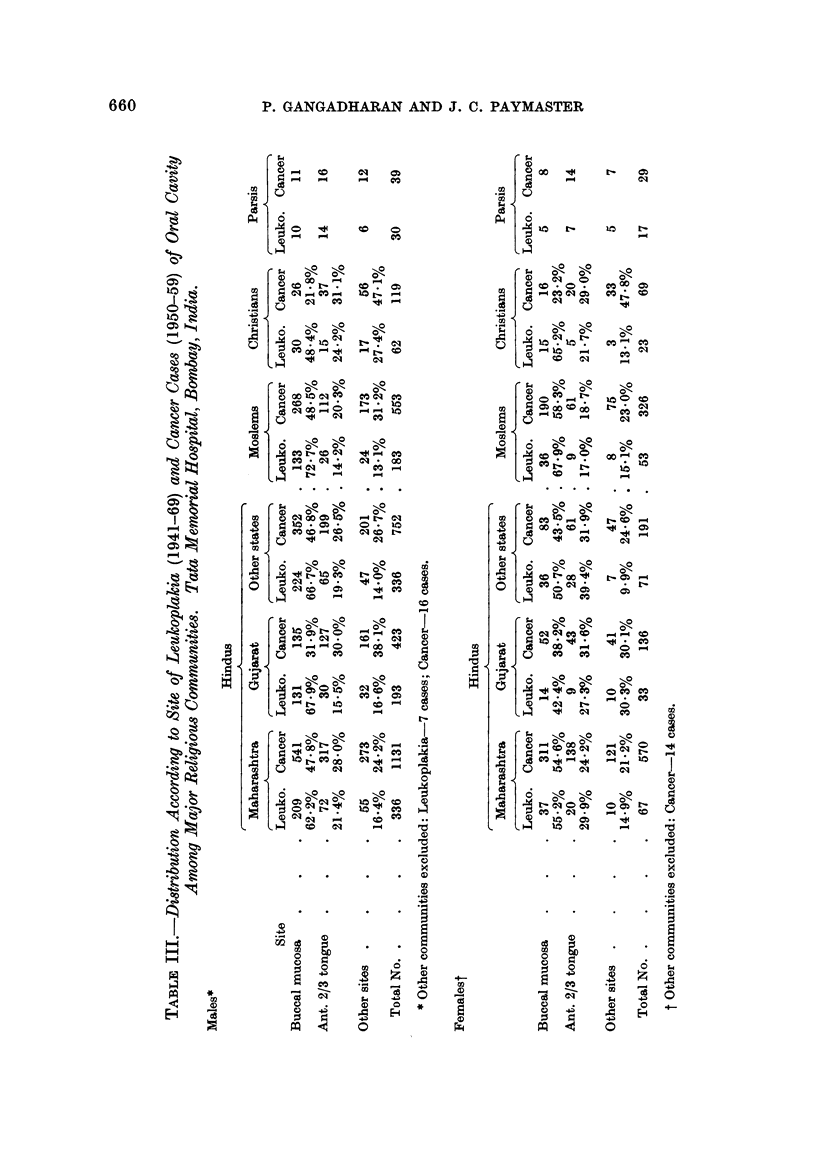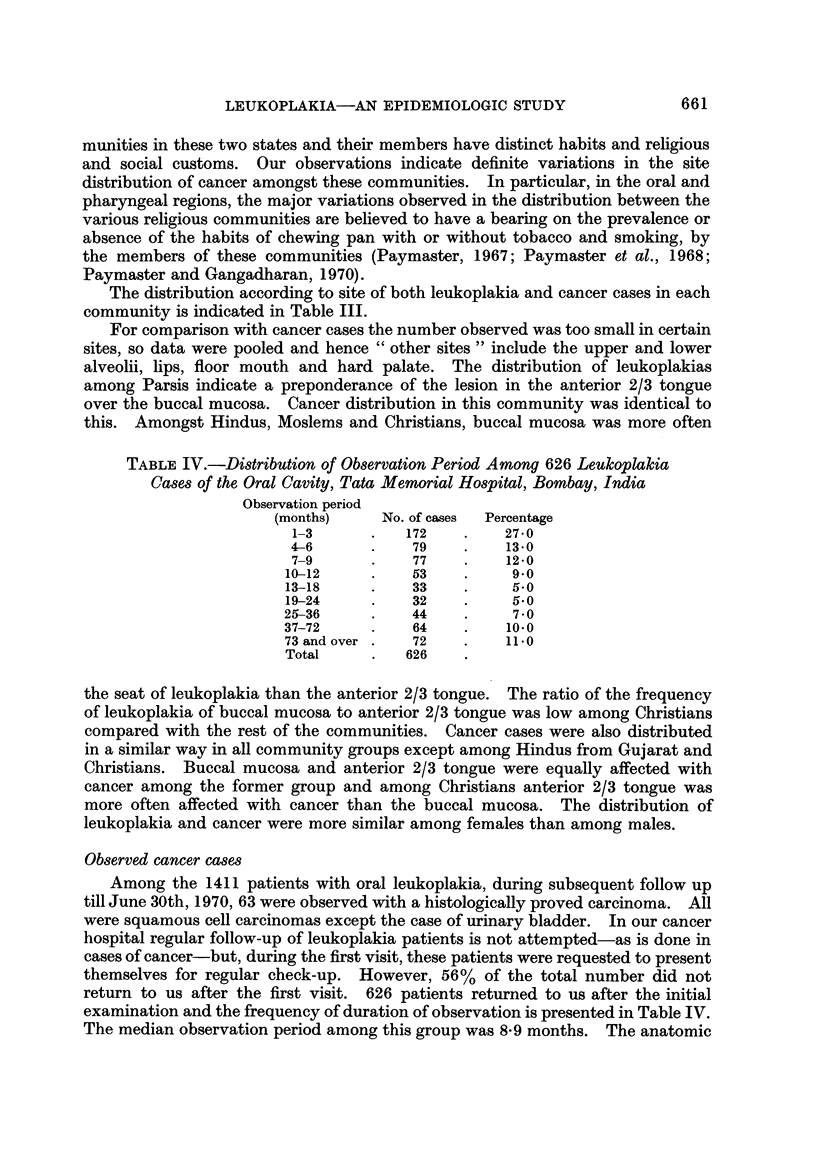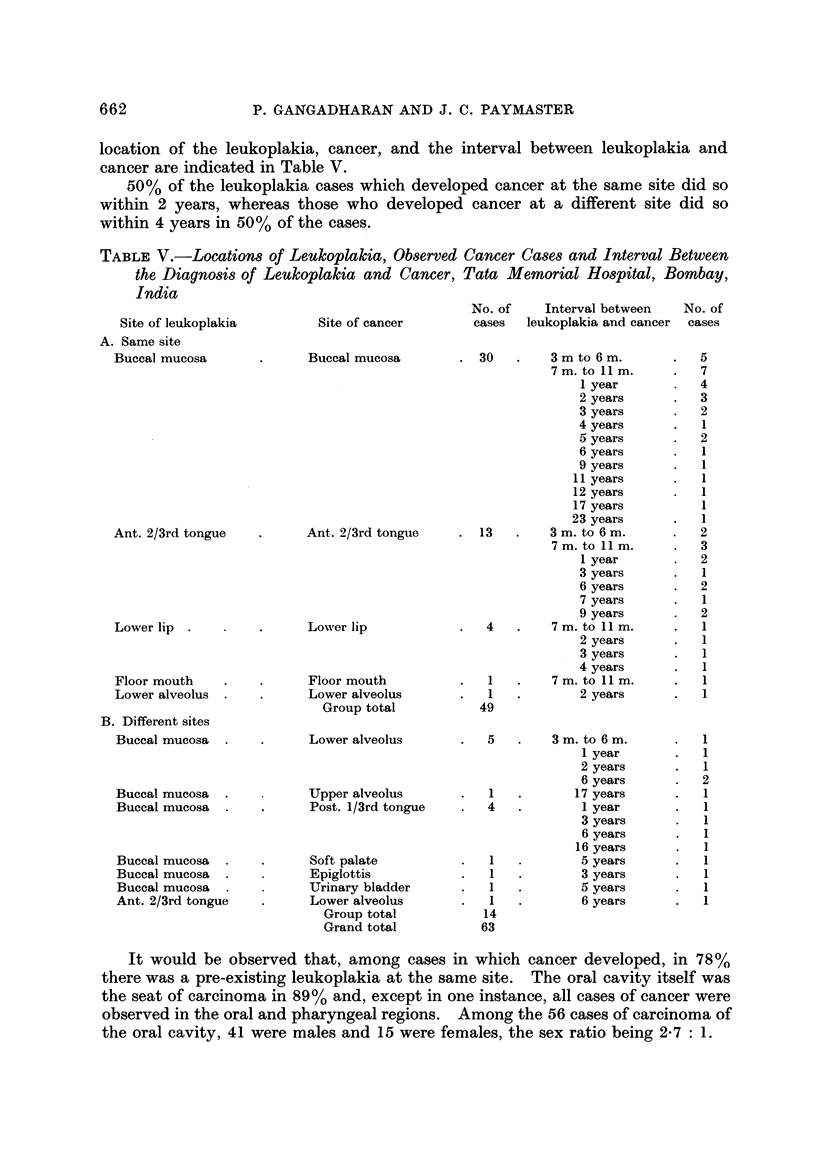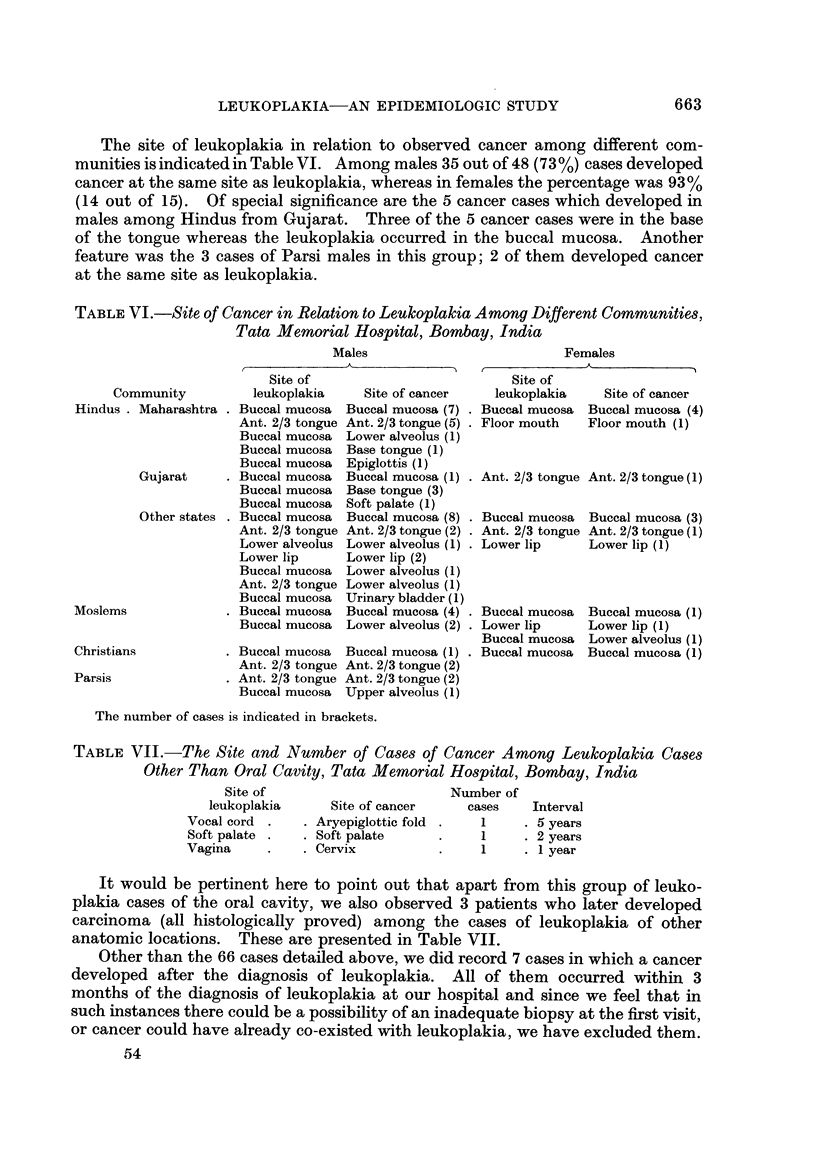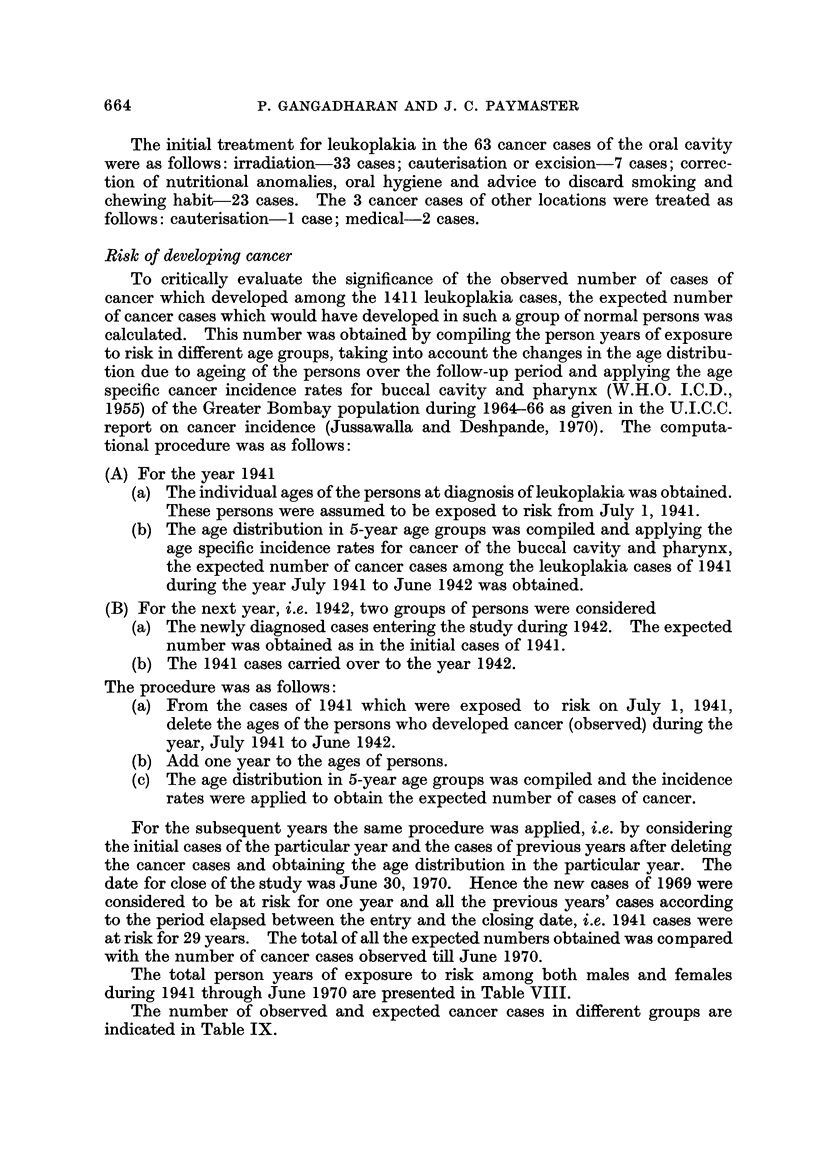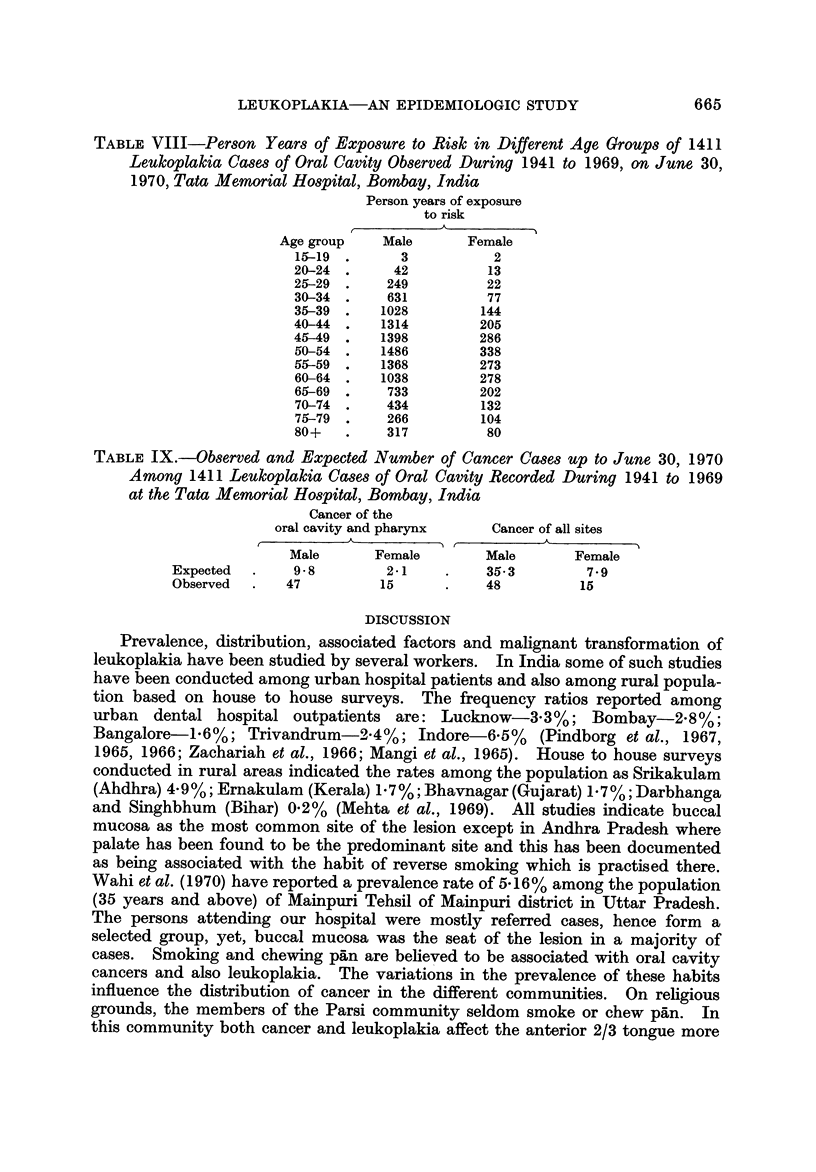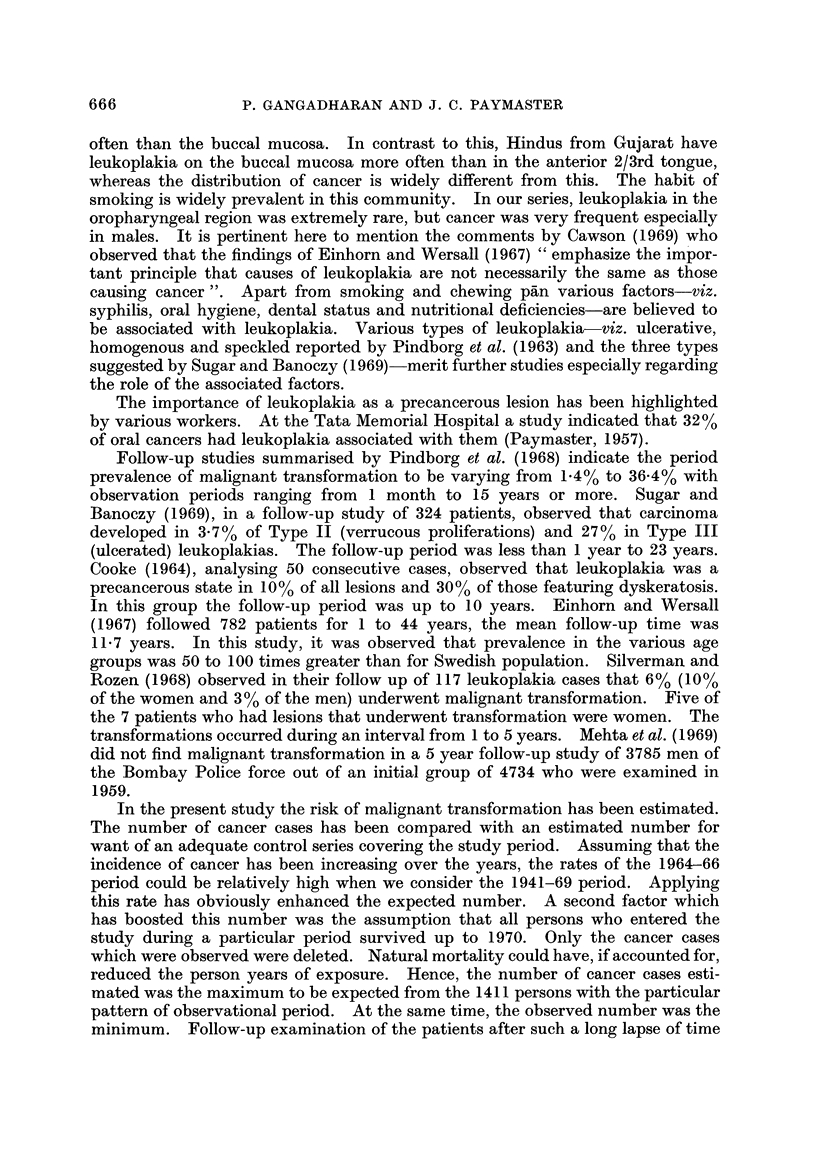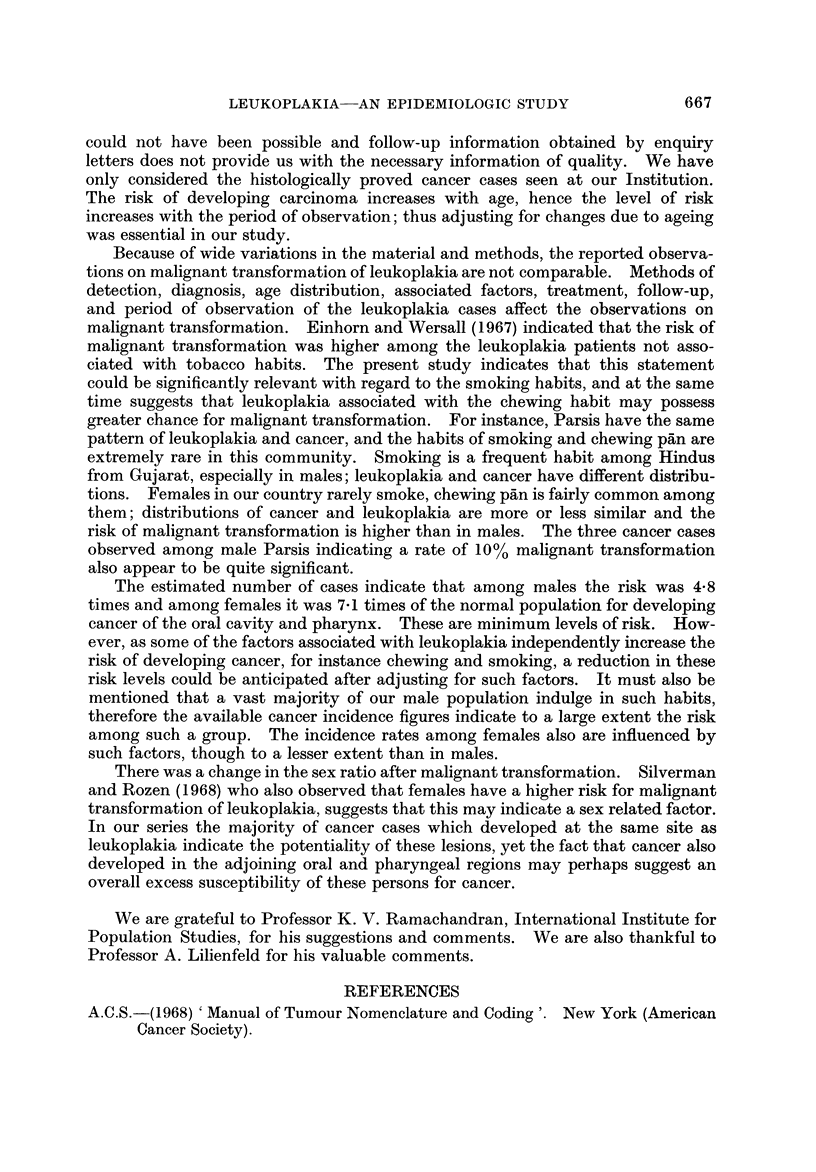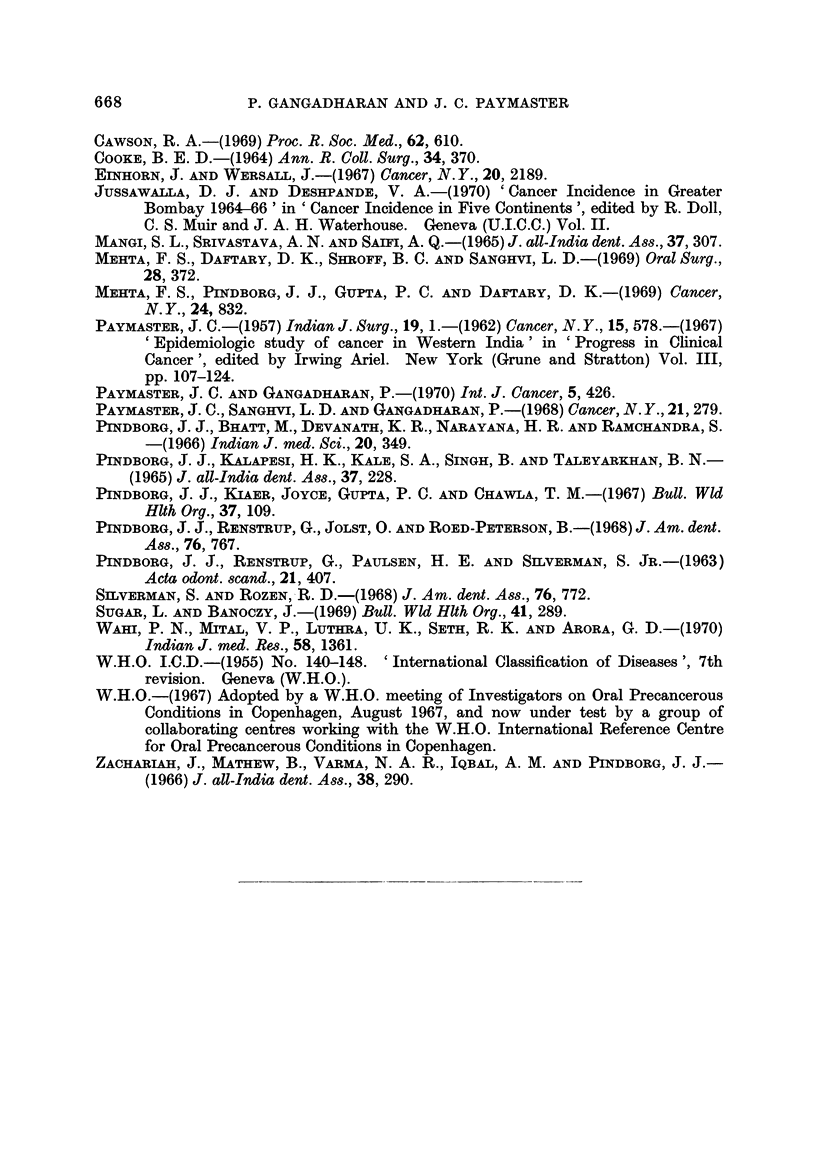# Leukoplakia—An Epidemiologic Study of 1504 Cases Observed at the Tata Memorial Hospital, Bombay, India

**DOI:** 10.1038/bjc.1971.81

**Published:** 1971-12

**Authors:** P. Gangadharan, J. C. Paymaster

## Abstract

An epidemiological study of 1504 cases of leukoplakia seen at the Tata Memorial Hospital, Bombay, indicates that the oral cavity was the site of the disease in 95% of the cases. The buccal mucosa was the commonest site affected in all religious communities of Western India except among Parsis. Parsis, a majority of whom are non-smokers and non-chewers of tobacco, had leukoplakia more often on the anterior 2/3rd tongue than on the buccal mucosa and this pattern persisted in the distribution of cancer also, whereas people from Gujarat more often smoke; in these the buccal mucosa was commonly affected with leukoplakia, but cancer was not so frequent in this site. Statistical computation of the risk of malignant transformation indicates that males have a 4·8 times higher risk of developing cancer when they have leukoplakia than the normal population, and the females have 7 times higher risk of developing cancer in the presence of leukoplakia. It was felt that leukoplakia not associated with smoking habits had a greater chance of malignant transformation.


### LEUKOPLAKIA—AN EPIDEMIOLOGIC STUDY OF 1504 CASES OBSERVED AT THE TATA MEMORIAL HOSPITAL, BOMBAY, INDIA

P. GANGADHARAN AND J. C. PAYMASTER

*From the Tata Memorial Centre, Dr. Ernest Borges Marg, Parel, Bombay-12, India*

Received for publication July 29, 1971

**SUMMARY.—An epidemiological study of 1504 cases of leukoplakia seen at the Tata Memorial Hospital, Bombay, indicates that the oral cavity was the site of the disease in 95% of the cases. The buccal mucosa was the commonest site affected in all religious communities of Western India except among Parsis. Parsis, a majority of whom are non-smokers and non-chewers of tobacco, had leukoplakia more often on the anterior 2/3rd tongue than on the buccal mucosa and this pattern persisted in the distribution of cancer also, whereas people from Gujarat more often smoke; in these the buccal mucosa was commonly affected with leukoplakia, but cancer was not so frequent in this site. Statistical computation of the risk of malignant transformation indicates that males have a 4·8 times higher risk of developing cancer when they have leukoplakia than the normal population, and the females have 7 times higher risk of developing cancer in the presence of leukoplakia. It was felt that leukoplakia not associated with smoking habits had a greater chance of malignant transformation.**

CERTAIN tissue changes occurring in the body have long been identified as precursors of cancer. Prominent among them is the leukoplakia arising in the oral cavity which is recognised as an intermediate stage in the progressive development of squamous cell carcinoma in a normal mucosa. However, not all squamous cell carcinomas of the oral cavity are preceded by a leukoplakia nor all leukoplakias undergo malignant transformation. The majority of these lesions are considered to be reversible or curable; yet, recurrences are observed in some cases.

Leukoplakia (of the oral cavity) has been defined as a white patch of the oral mucosa measuring 5 mm or more which cannot be scraped off and which cannot be attributed to any other diagnostic disease (W.H.O., 1967). 1504 cases of clinically diagnosed leukoplakia of all sites have been recorded at the Tata Memorial Hospital, Bombay, India, during a period of 29 years, 1941 to 1969, thus forming 0·7% of the total attendance of 203,249 persons during the same period.

Oral and pharyngeal carcinomas constitute 45·6% of all malignant neoplasms observed at our hospital. Our observations since 1941 reveal that these carcinomas frequently exhibit basic differences from one anatomic site to another. These include the biological characteristics, *viz.* rate of growth, local and distal spread, response to therapy, prognosis, end results and also the epidemiological features, *viz.* frequency, sex ratio and distribution in various religious communities (Paymaster, 1957, 1962). Thus we recognise oral and pharyngeal carcinomas in the following distinct anatomic zones: (1) the oral cavity (1400;

1401; 1411, 13, 14, 15; 1430; 1431; 1449; 1450; 1452), (2) the oropharynx (1410; 1453; 1461; 1466, 67), (3) the hypopharynx (1480; 1481; 1482; 1611), (4) the nasopharynx (147). The numbers in brackets indicate the code adopted for the sites included in the groups by American Cancer Society (A.C.S., 1968). Distribution among all cases of carcinoma seen at the Tata Memorial Hospital during 1950–59 in oral cavity and pharynx indicate the following pattern. Males: oral cavity 24·3%, oropharynx 46·4%, hypopharynx 28·5% and nasopharynx 0·8%; and females: oral cavity 61·9%, oropharynx 20·1%, hypopharynx 17·0% and nasopharynx 1·0%.

TABLE I.—*Site Distribution of* 1504 *Leukoplakia Cases Recorded at the Tata Memorial Hospital, Bombay, India,* 1941 *to* 1969

| Site | Males | | Females | |
|---|---|---|---|---|
| | Number | Percentage | Number | Percentage |
| Oral cavity | 1155 | 96·3 | 267 | 87·5 |
| Oropharynx | 8 | 0·7 | 1 | 0·3 |
| Intrinsic larynx | 15 | 1·3 | 2 | 0·7 |
| Vulva, vagina | — | — | 33 | 10·8 |
| Penis | 8 | 0·7 | — | — |
| Other and unspecified | 13 | 1·0 | 2 | 0·7 |
| Total | 1199 | 100·0 | 305 | 100·0 |

TABLE II.—*Distribution According to Anatomic Location of Leukoplakia* (1941–69) *and Cancer Cases* (1950–59) *Within the Oral Cavity, Tata Memorial Hospital, Bombay, India*

| Site | Males | | Females | |
|---|---|---|---|---|
| | Leukoplakia 1941–69 % | Cancer 1950–59 % | Leukoplakia 1941–69 % | Cancer 1950–59 % |
| Oral cavity | | | | |
| Buccal mucosa | 65·1 | 44·1 | 54·0 | 50·0 |
| Ant. 2/3rd tongue | 19·5 | 26·8 | 29·5 | 25·5 |
| Lips | 3·0 | 3·2 | 2·3 | 3·3 |
| Hard palate | 1·7 | 5·3 | 3·8 | 3·7 |
| Floor mouth | 0·8 | 4·8 | 0·4 | 3·2 |
| Lower alveolus | 0·6 | 13·8 | 0·4 | 11·6 |
| Upper alveolus | 0·2 | 1·9 | — | 2·7 |
| Multiple sites† | 9·2 | — | 9·6 | — |
| Total No. of cases | 1147* | 3033 | 264* | 1335 |

* Non-Indians excluded: Males—8; Females—3.
† Excluding bilateral lesions of buccal mucosa.

*Site distribution*

A similar grouping has been adopted for leukoplakia cases and Table I indicates the distribution according to site. Amongst the leukoplakia cases, in almost 53% a biopsy was done to exclude malignancy at the first visit of the patient to the hospital.

The preponderance of these lesions in the oral cavity is obvious. Intrinsic larynx (vocal cord) was the seat of leukoplakia more often than the oropharynx in men. Amongst females, 10·8% of the cases were located in the vulva or vagina.

This paper presents features of oral leukoplakia cases and their comparison with cancer cases seen during 1950–59 at our Institution.

The percentage distribution according to site of leukoplakia and carcinoma within the oral cavity is presented in Table II.

The site distribution of the cases of leukoplakia and cancer was almost similar among women. However, the frequency of cancer in floor mouth and lower alveolus were higher than the percentage of cases of leukoplakia occurring in these two sites. The marked excess of leukoplakia cases of the buccal mucosa in males is significant. Among the 747 males with lesions on the buccal mucosa, 227 (30·6%) had lesions on the opposite buccal mucosa also, while out of 143 females 32 (22·4%) had such bilateral lesions.

Fig. 1.—Age distribution of leukoplakia (1941–69) and cancer cases (1950–59) of oral cavity—Tata Memorial Hospital, Bombay.

*Age and sex distribution*

The age distribution and mean ages at diagnosis of both leukoplakia and cancer are indicated in Fig. 1.

The average age for the leukoplakia cases among males was lower than the cancer cases as well as the average age for both leukoplakia and cancer cases among females. Among males, the difference between the mean ages of leukoplakia and cancer cases was statistically significant; $P < 0{\cdot}01$.

The male to female ratio of oral leukoplakia cases was 4·3 : 1, whereas for oral cancer the ratio was 2·3 : 1.

*Religious community distribution*

Maharashtra and Gujarat are two large and important States of Western India. Hindus, Moslems, Christians and Parsis are the major religious com-

TABLE III.—*Distribution According to Site of Leukoplakia (1941–69) and Cancer Cases (1950–59) of Oral Cavity Among Major Religious Communities. Tata Memorial Hospital, Bombay, India.*

Males*

| Site | Hindus: Maharashtra Leuko. | Hindus: Maharashtra Cancer | Hindus: Gujarat Leuko. | Hindus: Gujarat Cancer | Hindus: Other states Leuko. | Hindus: Other states Cancer | Moslems Leuko. | Moslems Cancer | Christians Leuko. | Christians Cancer | Parsis Leuko. | Parsis Cancer |
|---|---|---|---|---|---|---|---|---|---|---|---|---|
| Buccal mucosa | 209 | 541 | 131 | 135 | 224 | 352 | 133 | 268 | 30 | 26 | 10 | 11 |
| | 62·2% | 47·8% | 67·9% | 31·9% | 66·7% | 46·8% | 72·7% | 48·5% | 48·4% | 21·8% | | |
| Ant. 2/3 tongue | 72 | 317 | 30 | 127 | 65 | 199 | 26 | 112 | 15 | 37 | 14 | 16 |
| | 21·4% | 28·0% | 15·5% | 30·0% | 19·3% | 26·5% | 14·2% | 20·3% | 24·2% | 31·1% | | |
| Other sites | 55 | 273 | 32 | 161 | 47 | 201 | 24 | 173 | 17 | 56 | 6 | 12 |
| | 16·4% | 24·2% | 16·6% | 38·1% | 14·0% | 26·7% | 13·1% | 31·2% | 27·4% | 47·1% | | |
| Total No. | 336 | 1131 | 193 | 423 | 336 | 752 | 183 | 553 | 62 | 119 | 30 | 39 |

* Other communities excluded: Leukoplakia—7 cases; Cancer—16 cases.

Females†

| Site | Hindus: Maharashtra Leuko. | Hindus: Maharashtra Cancer | Hindus: Gujarat Leuko. | Hindus: Gujarat Cancer | Hindus: Other states Leuko. | Hindus: Other states Cancer | Moslems Leuko. | Moslems Cancer | Christians Leuko. | Christians Cancer | Parsis Leuko. | Parsis Cancer |
|---|---|---|---|---|---|---|---|---|---|---|---|---|
| Buccal mucosa | 37 | 311 | 14 | 52 | 36 | 83 | 36 | 190 | 15 | 16 | 5 | 8 |
| | 55·2% | 54·6% | 42·4% | 38·2% | 50·7% | 43·5% | 67·9% | 58·3% | 65·2% | 23·2% | | |
| Ant. 2/3 tongue | 20 | 138 | 9 | 43 | 28 | 61 | 9 | 61 | 5 | 20 | 7 | 14 |
| | 29·9% | 24·2% | 27·3% | 31·6% | 39·4% | 31·9% | 17·0% | 18·7% | 21·7% | 29·0% | | |
| Other sites | 10 | 121 | 10 | 41 | 7 | 47 | 8 | 75 | 3 | 33 | 5 | 7 |
| | 14·9% | 21·2% | 30·3% | 30·1% | 9·9% | 24·6% | 15·1% | 23·0% | 13·1% | 47·8% | | |
| Total No. | 67 | 570 | 33 | 136 | 71 | 191 | 53 | 326 | 23 | 69 | 17 | 29 |

† Other communities excluded: Cancer—14 cases.

munities in these two states and their members have distinct habits and religious and social customs. Our observations indicate definite variations in the site distribution of cancer amongst these communities. In particular, in the oral and pharyngeal regions, the major variations observed in the distribution between the various religious communities are believed to have a bearing on the prevalence or absence of the habits of chewing pan with or without tobacco and smoking, by the members of these communities (Paymaster, 1967; Paymaster *et al.*, 1968; Paymaster and Gangadharan, 1970).

The distribution according to site of both leukoplakia and cancer cases in each community is indicated in Table III.

For comparison with cancer cases the number observed was too small in certain sites, so data were pooled and hence " other sites " include the upper and lower alveolii, lips, floor mouth and hard palate. The distribution of leukoplakias among Parsis indicate a preponderance of the lesion in the anterior 2/3 tongue over the buccal mucosa. Cancer distribution in this community was identical to this. Amongst Hindus, Moslems and Christians, buccal mucosa was more often

TABLE IV.—*Distribution of Observation Period Among* 626 *Leukoplakia Cases of the Oral Cavity, Tata Memorial Hospital, Bombay, India*

| Observation period (months) | No. of cases | Percentage |
|---|---|---|
| 1–3 | 172 | 27·0 |
| 4–6 | 79 | 13·0 |
| 7–9 | 77 | 12·0 |
| 10–12 | 53 | 9·0 |
| 13–18 | 33 | 5·0 |
| 19–24 | 32 | 5·0 |
| 25–36 | 44 | 7·0 |
| 37–72 | 64 | 10·0 |
| 73 and over | 72 | 11·0 |
| Total | 626 | |

the seat of leukoplakia than the anterior 2/3 tongue. The ratio of the frequency of leukoplakia of buccal mucosa to anterior 2/3 tongue was low among Christians compared with the rest of the communities. Cancer cases were also distributed in a similar way in all community groups except among Hindus from Gujarat and Christians. Buccal mucosa and anterior 2/3 tongue were equally affected with cancer among the former group and among Christians anterior 2/3 tongue was more often affected with cancer than the buccal mucosa. The distribution of leukoplakia and cancer were more similar among females than among males.

*Observed cancer cases*

Among the 1411 patients with oral leukoplakia, during subsequent follow up till June 30th, 1970, 63 were observed with a histologically proved carcinoma. All were squamous cell carcinomas except the case of urinary bladder. In our cancer hospital regular follow-up of leukoplakia patients is not attempted—as is done in cases of cancer—but, during the first visit, these patients were requested to present themselves for regular check-up. However, 56% of the total number did not return to us after the first visit. 626 patients returned to us after the initial examination and the frequency of duration of observation is presented in Table IV. The median observation period among this group was 8·9 months. The anatomic location of the leukoplakia, cancer, and the interval between leukoplakia and cancer are indicated in Table V.

50% of the leukoplakia cases which developed cancer at the same site did so within 2 years, whereas those who developed cancer at a different site did so within 4 years in 50% of the cases.

TABLE V.—*Locations of Leukoplakia, Observed Cancer Cases and Interval Between the Diagnosis of Leukoplakia and Cancer, Tata Memorial Hospital, Bombay, India*

| Site of leukoplakia | Site of cancer | No. of cases | Interval between leukoplakia and cancer | No. of cases |
|---|---|---|---|---|
| A. Same site | | | | |
| Buccal mucosa | Buccal mucosa | 30 | 3 m to 6 m. | 5 |
| | | | 7 m. to 11 m. | 7 |
| | | | 1 year | 4 |
| | | | 2 years | 3 |
| | | | 3 years | 2 |
| | | | 4 years | 1 |
| | | | 5 years | 2 |
| | | | 6 years | 1 |
| | | | 9 years | 1 |
| | | | 11 years | 1 |
| | | | 12 years | 1 |
| | | | 17 years | 1 |
| | | | 23 years | 1 |
| Ant. 2/3rd tongue | Ant. 2/3rd tongue | 13 | 3 m. to 6 m. | 2 |
| | | | 7 m. to 11 m. | 3 |
| | | | 1 year | 2 |
| | | | 3 years | 1 |
| | | | 6 years | 2 |
| | | | 7 years | 1 |
| | | | 9 years | 2 |
| Lower lip | Lower lip | 4 | 7 m. to 11 m. | 1 |
| | | | 2 years | 1 |
| | | | 3 years | 1 |
| | | | 4 years | 1 |
| Floor mouth | Floor mouth | 1 | 7 m. to 11 m. | 1 |
| Lower alveolus | Lower alveolus | 1 | 2 years | 1 |
| | Group total | 49 | | |
| B. Different sites | | | | |
| Buccal mucosa | Lower alveolus | 5 | 3 m. to 6 m. | 1 |
| | | | 1 year | 1 |
| | | | 2 years | 1 |
| | | | 6 years | 2 |
| Buccal mucosa | Upper alveolus | 1 | 17 years | 1 |
| Buccal mucosa | Post. 1/3rd tongue | 4 | 1 year | 1 |
| | | | 3 years | 1 |
| | | | 6 years | 1 |
| | | | 16 years | 1 |
| Buccal mucosa | Soft palate | 1 | 5 years | 1 |
| Buccal mucosa | Epiglottis | 1 | 3 years | 1 |
| Buccal mucosa | Urinary bladder | 1 | 5 years | 1 |
| Ant. 2/3rd tongue | Lower alveolus | 1 | 6 years | 1 |
| | Group total | 14 | | |
| | Grand total | 63 | | |

It would be observed that, among cases in which cancer developed, in 78% there was a pre-existing leukoplakia at the same site. The oral cavity itself was the seat of carcinoma in 89% and, except in one instance, all cases of cancer were observed in the oral and pharyngeal regions. Among the 56 cases of carcinoma of the oral cavity, 41 were males and 15 were females, the sex ratio being 2·7 : 1.

The site of leukoplakia in relation to observed cancer among different communities is indicated in Table VI. Among males 35 out of 48 (73%) cases developed cancer at the same site as leukoplakia, whereas in females the percentage was 93% (14 out of 15). Of special significance are the 5 cancer cases which developed in males among Hindus from Gujarat. Three of the 5 cancer cases were in the base of the tongue whereas the leukoplakia occurred in the buccal mucosa. Another feature was the 3 cases of Parsi males in this group; 2 of them developed cancer at the same site as leukoplakia.

TABLE VI.—*Site of Cancer in Relation to Leukoplakia Among Different Communities, Tata Memorial Hospital, Bombay, India*

| Community | | Males | | Females | |
|---|---|---|---|---|---|
| | | Site of leukoplakia | Site of cancer | Site of leukoplakia | Site of cancer |
| Hindus | Maharashtra | Buccal mucosa | Buccal mucosa (7) | Buccal mucosa | Buccal mucosa (4) |
| | | Ant. 2/3 tongue | Ant. 2/3 tongue (5) | Floor mouth | Floor mouth (1) |
| | | Buccal mucosa | Lower alveolus (1) | | |
| | | Buccal mucosa | Base tongue (1) | | |
| | | Buccal mucosa | Epiglottis (1) | | |
| | Gujarat | Buccal mucosa | Buccal mucosa (1) | Ant. 2/3 tongue | Ant. 2/3 tongue (1) |
| | | Buccal mucosa | Base tongue (3) | | |
| | | Buccal mucosa | Soft palate (1) | | |
| | Other states | Buccal mucosa | Buccal mucosa (8) | Buccal mucosa | Buccal mucosa (3) |
| | | Ant. 2/3 tongue | Ant. 2/3 tongue (2) | Ant. 2/3 tongue | Ant. 2/3 tongue (1) |
| | | Lower alveolus | Lower alveolus (1) | Lower lip | Lower lip (1) |
| | | Lower lip | Lower lip (2) | | |
| | | Buccal mucosa | Lower alveolus (1) | | |
| | | Ant. 2/3 tongue | Lower alveolus (1) | | |
| | | Buccal mucosa | Urinary bladder (1) | | |
| Moslems | | Buccal mucosa | Buccal mucosa (4) | Buccal mucosa | Buccal mucosa (1) |
| | | Buccal mucosa | Lower alveolus (2) | Lower lip | Lower lip (1) |
| | | | | Buccal mucosa | Lower alveolus (1) |
| Christians | | Buccal mucosa | Buccal mucosa (1) | Buccal mucosa | Buccal mucosa (1) |
| | | Ant. 2/3 tongue | Ant. 2/3 tongue (2) | | |
| Parsis | | Ant. 2/3 tongue | Ant. 2/3 tongue (2) | | |
| | | Buccal mucosa | Upper alveolus (1) | | |

The number of cases is indicated in brackets.

TABLE VII.—*The Site and Number of Cases of Cancer Among Leukoplakia Cases Other Than Oral Cavity, Tata Memorial Hospital, Bombay, India*

| Site of leukoplakia | Site of cancer | Number of cases | Interval |
|---|---|---|---|
| Vocal cord | Aryepiglottic fold | 1 | 5 years |
| Soft palate | Soft palate | 1 | 2 years |
| Vagina | Cervix | 1 | 1 year |

It would be pertinent here to point out that apart from this group of leukoplakia cases of the oral cavity, we also observed 3 patients who later developed carcinoma (all histologically proved) among the cases of leukoplakia of other anatomic locations. These are presented in Table VII.

Other than the 66 cases detailed above, we did record 7 cases in which a cancer developed after the diagnosis of leukoplakia. All of them occurred within 3 months of the diagnosis of leukoplakia at our hospital and since we feel that in such instances there could be a possibility of an inadequate biopsy at the first visit, or cancer could have already co-existed with leukoplakia, we have excluded them.

The initial treatment for leukoplakia in the 63 cancer cases of the oral cavity were as follows: irradiation—33 cases; cauterisation or excision—7 cases; correction of nutritional anomalies, oral hygiene and advice to discard smoking and chewing habit—23 cases. The 3 cancer cases of other locations were treated as follows: cauterisation—1 case; medical—2 cases.

*Risk of developing cancer*

To critically evaluate the significance of the observed number of cases of cancer which developed among the 1411 leukoplakia cases, the expected number of cancer cases which would have developed in such a group of normal persons was calculated. This number was obtained by compiling the person years of exposure to risk in different age groups, taking into account the changes in the age distribution due to ageing of the persons over the follow-up period and applying the age specific cancer incidence rates for buccal cavity and pharynx (W.H.O. I.C.D., 1955) of the Greater Bombay population during 1964–66 as given in the U.I.C.C. report on cancer incidence (Jussawalla and Deshpande, 1970). The computational procedure was as follows:

(A) For the year 1941
- (a) The individual ages of the persons at diagnosis of leukoplakia was obtained. These persons were assumed to be exposed to risk from July 1, 1941.
- (b) The age distribution in 5-year age groups was compiled and applying the age specific incidence rates for cancer of the buccal cavity and pharynx, the expected number of cancer cases among the leukoplakia cases of 1941 during the year July 1941 to June 1942 was obtained.

(B) For the next year, *i.e.* 1942, two groups of persons were considered
- (a) The newly diagnosed cases entering the study during 1942. The expected number was obtained as in the initial cases of 1941.
- (b) The 1941 cases carried over to the year 1942.

The procedure was as follows:
- (a) From the cases of 1941 which were exposed to risk on July 1, 1941, delete the ages of the persons who developed cancer (observed) during the year, July 1941 to June 1942.
- (b) Add one year to the ages of persons.
- (c) The age distribution in 5-year age groups was compiled and the incidence rates were applied to obtain the expected number of cases of cancer.

For the subsequent years the same procedure was applied, *i.e.* by considering the initial cases of the particular year and the cases of previous years after deleting the cancer cases and obtaining the age distribution in the particular year. The date for close of the study was June 30, 1970. Hence the new cases of 1969 were considered to be at risk for one year and all the previous years' cases according to the period elapsed between the entry and the closing date, *i.e.* 1941 cases were at risk for 29 years. The total of all the expected numbers obtained was compared with the number of cancer cases observed till June 1970.

The total person years of exposure to risk among both males and females during 1941 through June 1970 are presented in Table VIII.

The number of observed and expected cancer cases in different groups are indicated in Table IX.

TABLE VIII—*Person Years of Exposure to Risk in Different Age Groups of* 1411 *Leukoplakia Cases of Oral Cavity Observed During* 1941 *to* 1969, *on June* 30, 1970, *Tata Memorial Hospital, Bombay, India*

| Age group | Person years of exposure to risk: Male | Female |
|---|---|---|
| 15–19 | 3 | 2 |
| 20–24 | 42 | 13 |
| 25–29 | 249 | 22 |
| 30–34 | 631 | 77 |
| 35–39 | 1028 | 144 |
| 40–44 | 1314 | 205 |
| 45–49 | 1398 | 286 |
| 50–54 | 1486 | 338 |
| 55–59 | 1368 | 273 |
| 60–64 | 1038 | 278 |
| 65–69 | 733 | 202 |
| 70–74 | 434 | 132 |
| 75–79 | 266 | 104 |
| 80+ | 317 | 80 |

TABLE IX.—*Observed and Expected Number of Cancer Cases up to June* 30, 1970 *Among* 1411 *Leukoplakia Cases of Oral Cavity Recorded During* 1941 *to* 1969 *at the Tata Memorial Hospital, Bombay, India*

| | Cancer of the oral cavity and pharynx | | Cancer of all sites | |
|---|---|---|---|---|
| | Male | Female | Male | Female |
| Expected | 9·8 | 2·1 | 35·3 | 7·9 |
| Observed | 47 | 15 | 48 | 15 |

### DISCUSSION

Prevalence, distribution, associated factors and malignant transformation of leukoplakia have been studied by several workers. In India some of such studies have been conducted among urban hospital patients and also among rural population based on house to house surveys. The frequency ratios reported among urban dental hospital outpatients are: Lucknow—3·3%; Bombay—2·8%; Bangalore—1·6%; Trivandrum—2·4%; Indore—6·5% (Pindborg *et al.*, 1967, 1965, 1966; Zachariah *et al.*, 1966; Mangi *et al.*, 1965). House to house surveys conducted in rural areas indicated the rates among the population as Srikakulam (Ahdhra) 4·9%; Ernakulam (Kerala) 1·7%; Bhavnagar (Gujarat) 1·7%; Darbhanga and Singhbhum (Bihar) 0·2% (Mehta *et al.*, 1969). All studies indicate buccal mucosa as the most common site of the lesion except in Andhra Pradesh where palate has been found to be the predominant site and this has been documented as being associated with the habit of reverse smoking which is practised there. Wahi *et al.* (1970) have reported a prevalence rate of 5·16% among the population (35 years and above) of Mainpuri Tehsil of Mainpuri district in Uttar Pradesh. The persons attending our hospital were mostly referred cases, hence form a selected group, yet, buccal mucosa was the seat of the lesion in a majority of cases. Smoking and chewing pān are believed to be associated with oral cavity cancers and also leukoplakia. The variations in the prevalence of these habits influence the distribution of cancer in the different communities. On religious grounds, the members of the Parsi community seldom smoke or chew pān. In this community both cancer and leukoplakia affect the anterior 2/3 tongue more often than the buccal mucosa. In contrast to this, Hindus from Gujarat have leukoplakia on the buccal mucosa more often than in the anterior 2/3rd tongue, whereas the distribution of cancer is widely different from this. The habit of smoking is widely prevalent in this community. In our series, leukoplakia in the oropharyngeal region was extremely rare, but cancer was very frequent especially in males. It is pertinent here to mention the comments by Cawson (1969) who observed that the findings of Einhorn and Wersall (1967) " emphasize the important principle that causes of leukoplakia are not necessarily the same as those causing cancer ". Apart from smoking and chewing pān various factors—*viz.* syphilis, oral hygiene, dental status and nutritional deficiencies—are believed to be associated with leukoplakia. Various types of leukoplakia—*viz.* ulcerative, homogenous and speckled reported by Pindborg *et al.* (1963) and the three types suggested by Sugar and Banoczy (1969)—merit further studies especially regarding the role of the associated factors.

The importance of leukoplakia as a precancerous lesion has been highlighted by various workers. At the Tata Memorial Hospital a study indicated that 32% of oral cancers had leukoplakia associated with them (Paymaster, 1957).

Follow-up studies summarised by Pindborg *et al.* (1968) indicate the period prevalence of malignant transformation to be varying from 1·4% to 36·4% with observation periods ranging from 1 month to 15 years or more. Sugar and Banoczy (1969), in a follow-up study of 324 patients, observed that carcinoma developed in 3·7% of Type II (verrucous proliferations) and 27% in Type III (ulcerated) leukoplakias. The follow-up period was less than 1 year to 23 years. Cooke (1964), analysing 50 consecutive cases, observed that leukoplakia was a precancerous state in 10% of all lesions and 30% of those featuring dyskeratosis. In this group the follow-up period was up to 10 years. Einhorn and Wersall (1967) followed 782 patients for 1 to 44 years, the mean follow-up time was 11·7 years. In this study, it was observed that prevalence in the various age groups was 50 to 100 times greater than for Swedish population. Silverman and Rozen (1968) observed in their follow up of 117 leukoplakia cases that 6% (10% of the women and 3% of the men) underwent malignant transformation. Five of the 7 patients who had lesions that underwent transformation were women. The transformations occurred during an interval from 1 to 5 years. Mehta *et al.* (1969) did not find malignant transformation in a 5 year follow-up study of 3785 men of the Bombay Police force out of an initial group of 4734 who were examined in 1959.

In the present study the risk of malignant transformation has been estimated. The number of cancer cases has been compared with an estimated number for want of an adequate control series covering the study period. Assuming that the incidence of cancer has been increasing over the years, the rates of the 1964–66 period could be relatively high when we consider the 1941–69 period. Applying this rate has obviously enhanced the expected number. A second factor which has boosted this number was the assumption that all persons who entered the study during a particular period survived up to 1970. Only the cancer cases which were observed were deleted. Natural mortality could have, if accounted for, reduced the person years of exposure. Hence, the number of cancer cases estimated was the maximum to be expected from the 1411 persons with the particular pattern of observational period. At the same time, the observed number was the minimum. Follow-up examination of the patients after such a long lapse of time could not have been possible and follow-up information obtained by enquiry letters does not provide us with the necessary information of quality. We have only considered the histologically proved cancer cases seen at our Institution. The risk of developing carcinoma increases with age, hence the level of risk increases with the period of observation; thus adjusting for changes due to ageing was essential in our study.

Because of wide variations in the material and methods, the reported observations on malignant transformation of leukoplakia are not comparable. Methods of detection, diagnosis, age distribution, associated factors, treatment, follow-up, and period of observation of the leukoplakia cases affect the observations on malignant transformation. Einhorn and Wersall (1967) indicated that the risk of malignant transformation was higher among the leukoplakia patients not associated with tobacco habits. The present study indicates that this statement could be significantly relevant with regard to the smoking habits, and at the same time suggests that leukoplakia associated with the chewing habit may possess greater chance for malignant transformation. For instance, Parsis have the same pattern of leukoplakia and cancer, and the habits of smoking and chewing pān are extremely rare in this community. Smoking is a frequent habit among Hindus from Gujarat, especially in males; leukoplakia and cancer have different distributions. Females in our country rarely smoke, chewing pān is fairly common among them; distributions of cancer and leukoplakia are more or less similar and the risk of malignant transformation is higher than in males. The three cancer cases observed among male Parsis indicating a rate of 10% malignant transformation also appear to be quite significant.

The estimated number of cases indicate that among males the risk was 4·8 times and among females it was 7·1 times of the normal population for developing cancer of the oral cavity and pharynx. These are minimum levels of risk. However, as some of the factors associated with leukoplakia independently increase the risk of developing cancer, for instance chewing and smoking, a reduction in these risk levels could be anticipated after adjusting for such factors. It must also be mentioned that a vast majority of our male population indulge in such habits, therefore the available cancer incidence figures indicate to a large extent the risk among such a group. The incidence rates among females also are influenced by such factors, though to a lesser extent than in males.

There was a change in the sex ratio after malignant transformation. Silverman and Rozen (1968) who also observed that females have a higher risk for malignant transformation of leukoplakia, suggests that this may indicate a sex related factor. In our series the majority of cancer cases which developed at the same site as leukoplakia indicate the potentiality of these lesions, yet the fact that cancer also developed in the adjoining oral and pharyngeal regions may perhaps suggest an overall excess susceptibility of these persons for cancer.

We are grateful to Professor K. V. Ramachandran, International Institute for Population Studies, for his suggestions and comments. We are also thankful to Professor A. Lilienfeld for his valuable comments.

### REFERENCES

A.C.S.—(1968) ‘ Manual of Tumour Nomenclature and Coding ’. New York (American Cancer Society).

CAWSON, R. A.—(1969) *Proc. R. Soc. Med.*, **62**, 610.
COOKE, B. E. D.—(1964) *Ann. R. Coll. Surg.*, **34**, 370.
EINHORN, J. AND WERSALL, J.—(1967) *Cancer, N.Y.*, **20**, 2189.
JUSSAWALLA, D. J. AND DESHPANDE, V. A.—(1970) 'Cancer Incidence in Greater Bombay 1964–66' in 'Cancer Incidence in Five Continents', edited by R. Doll, C. S. Muir and J. A. H. Waterhouse. Geneva (U.I.C.C.) Vol. II.
MANGI, S. L., SRIVASTAVA, A. N. AND SAIFI, A. Q.—(1965) *J. all-India dent. Ass.*, **37**, 307.
MEHTA, F. S., DAFTARY, D. K., SHROFF, B. C. AND SANGHVI, L. D.—(1969) *Oral Surg.*, **28**, 372.
MEHTA, F. S., PINDBORG, J. J., GUPTA, P. C. AND DAFTARY, D. K.—(1969) *Cancer, N.Y.*, **24**, 832.
PAYMASTER, J. C.—(1957) *Indian J. Surg.*, **19**, 1.—(1962) *Cancer, N.Y.*, **15**, 578.—(1967) 'Epidemiologic study of cancer in Western India' in 'Progress in Clinical Cancer', edited by Irwing Ariel. New York (Grune and Stratton) Vol. III, pp. 107–124.
PAYMASTER, J. C. AND GANGADHARAN, P.—(1970) *Int. J. Cancer*, **5**, 426.
PAYMASTER, J. C., SANGHVI, L. D. AND GANGADHARAN, P.—(1968) *Cancer, N.Y.*, **21**, 279.
PINDBORG, J. J., BHATT, M., DEVANATH, K. R., NARAYANA, H. R. AND RAMCHANDRA, S. —(1966) *Indian J. med. Sci.*, **20**, 349.
PINDBORG, J. J., KALAPESI, H. K., KALE, S. A., SINGH, B. AND TALEYARKHAN, B. N.—(1965) *J. all-India dent. Ass.*, **37**, 228.
PINDBORG, J. J., KIAER, JOYCE, GUPTA, P. C. AND CHAWLA, T. M.—(1967) *Bull. Wld Hlth Org.*, **37**, 109.
PINDBORG, J. J., RENSTRUP, G., JOLST, O. AND ROED-PETERSON, B.—(1968) *J. Am. dent. Ass.*, **76**, 767.
PINDBORG, J. J., RENSTRUP, G., PAULSEN, H. E. AND SILVERMAN, S. JR.—(1963) *Acta odont. scand.*, **21**, 407.
SILVERMAN, S. AND ROZEN, R. D.—(1968) *J. Am. dent. Ass.*, **76**, 772.
SUGAR, L. AND BANOCZY, J.—(1969) *Bull. Wld Hlth Org.*, **41**, 289.
WAHI, P. N., MITAL, V. P., LUTHRA, U. K., SETH, R. K. AND ARORA, G. D.—(1970) *Indian J. med. Res.*, **58**, 1361.
W.H.O. I.C.D.—(1955) No. 140–148. 'International Classification of Diseases', 7th revision. Geneva (W.H.O.).
W.H.O.—(1967) Adopted by a W.H.O. meeting of Investigators on Oral Precancerous Conditions in Copenhagen, August 1967, and now under test by a group of collaborating centres working with the W.H.O. International Reference Centre for Oral Precancerous Conditions in Copenhagen.
ZACHARIAH, J., MATHEW, B., VARMA, N. A. R., IQBAL, A. M. AND PINDBORG, J. J.—(1966) *J. all-India dent. Ass.*, **38**, 290.